# Visitors to a Tropical Marine Beach Show Evidence of Immunoconversions to Multiple Waterborne Pathogens

**DOI:** 10.3389/fpubh.2019.00231

**Published:** 2019-08-19

**Authors:** Kaneatra J. Simmons, Tarsha N. Eason, Clarissa L. Curioso, Shannon M. Griffin, Malini K. D. Ramudit, Kevin H. Oshima, Elizabeth A. Sams, Timothy J. Wade, Ann Grimm, Alfred Dufour, Swinburne A. J. Augustine

**Affiliations:** ^1^Department of Arts & Sciences/Learning Support, Fort Valley State University, Fort Valley, GA, United States; ^2^National Risk Management Research Laboratory, U.S. Environmental Protection Agency, Cincinnati, OH, United States; ^3^Oak Ridge Institute for Science Education, Oak Ridge, TN, United States; ^4^National Exposure Research Laboratory, U.S. Environmental Protection Agency, Cincinnati, OH, United States; ^5^National Health and Environmental Effects Research Laboratory, Research Triangle Park, NC, United States

**Keywords:** immunoconversions, incident infections, exposure, multiplex immunoassay, salivary antibodies, antibody response, Luminex, Boquerón Beach

## Abstract

Determining infections from environmental exposures, particularly from waterborne pathogens is a challenging proposition. The study design must be rigorous and account for numerous factors including study population selection, sample collection, storage, and processing, as well as data processing and analysis. These challenges are magnified when it is suspected that individuals may potentially be infected by multiple pathogens at the same time. Previous work demonstrated the effectiveness of a salivary antibody multiplex immunoassay in detecting the prevalence of immunoglobulin G (IgG) antibodies to multiple waterborne pathogens and helped identify asymptomatic norovirus infections in visitors to Boquerón Beach, Puerto Rico. In this study, we applied the immunoassay to three serially collected samples from study participants within the same population to assess immunoconversions (incident infections) to six waterborne pathogens: *Helicobacter pylori, Campylobacter jejuni, Toxoplasma gondii*, hepatitis A virus, and noroviruses GI. I and GII.4. Further, we examined the impact of sampling on the detection of immunoconversions by comparing the traditional immunoconversion definition based on two samples to criteria developed to capture trends in three sequential samples collected from study participants. The expansion to three samples makes it possible to capture the IgG antibody responses within the survey population to more accurately assess the frequency of immunoconversions to target pathogens. Based on the criteria developed, results showed that when only two samples from each participant were used in the analysis, 25.9% of the beachgoers immunoconverted to at least one pathogen; however, the addition of the third sample reduced immunoconversions to 6.5%. Of these incident infections, the highest levels were to noroviruses followed by *T. gondii*. Moreover, many individuals displayed evidence of immunoconversions to multiple pathogens. This study suggests that detection of simultaneous infections is possible, with far reaching consequences for the population. The results may lead to further studies to understand the complex interactions that occur within the body as the immune system attempts to ward off these infections. Such an approach is critical to our understanding of medically important synergistic or antagonistic interactions and may provide valuable and critical information to public health officials, water treatment personnel, and environmental managers.

## Introduction

Understanding the complex relationship between water quality, human exposure to waterborne pathogens, and subsequent infections is an ongoing public health challenge. Previous studies have shown considerable risk of illness related to polluted recreational and potable water (1–4). One of the primary risks associated with such exposures is the manifestation of acute gastrointestinal illness which may be caused by a variety of viruses, bacteria, and protozoa. In lieu of classic approaches to studying exposure (e.g., water sampling, risk modeling, epidemiological surveys, enzyme-linked immunosorbent assays), we developed a bead-based salivary IgG antibody multiplex immunoassay using the Luminex™ xMAP platform. Many traditional immunological studies involve examining antibodies in a monoplex, one pathogen at a time. Monoplex immunoassays, in general, do not truly capture the dynamic nature of the immune system and obscure our ability to observe simultaneous antibody responses to multiple pathogens. The multiplex developed and applied in this study assesses IgG antibodies in saliva to examine exposure to multiple pathogens simultaneously (5–7).

Saliva is a biofluid with emerging applications in research and clinical settings. We previously demonstrated that salivary IgG antibodies offer a viable alternative to the traditional use of blood, serum, and plasma for assessing immunoprevalence to waterborne pathogens (5, 8, 9). Researchers have noted the many benefits of using saliva including ease of collection, transport, and storage, applicability to remote collection and point-of-care settings, non-invasiveness, and the use of sampling mechanisms which are easily tolerable by children. The use of assays based on saliva are thus expected to have positive impacts on infant and child health (10–12).

We previously determined the prevalence of salivary antibodies against six waterborne pathogens: *Helicobacter pylori, Campylobacter jejuni, Toxoplasma gondii*, hepatitis A virus, and norovirus genogroups G.I.I and GII.4 in beachgoers and swimmers at Boquerón Beach, Puerto Rico using the multiplex immunoassay (5–7, 13, 14). We also linked epidemiological factors to the norovirus exposures and were able to identify asymptomatic infections (i.e., detectable infections unlinked to symptomology) associated with swimming at a fecally contaminated beach (15). As a follow-up, we aim to determine immunoconversions and evidence of multiple infections to the six target pathogens using samples collected from visitors to the beach which has been shown to be impacted by sewage from publicly-owned treatment works.

Immunoconversion relates to the development of detectable antibodies in the blood and saliva, usually within days after exposure to infectious agents. Soon after an initial exposure and during the acute phase of an infection, IgM antibodies along with other immune system components dominate the response. Later IgG antibodies that retain memory of the exposure are produced and these immunoglobulins rapidly expand in the event of a secondary infection as part of what has been described as the anamnestic response (16).

Previous immunoconversion studies relied on two serum samples (one pre-exposure and another post-exposure) obtained via invasive procedures. These studies measured a 4-fold increase in antibody response from the first to second sample to determine whether there was a change in antibody levels indicative of an active infection. In our study, we collected an initial sample (S1) at the beach before swimming, and the remaining samples (S2 and S3) were self-collected by participants 10–14 and 30–40 days later, respectively. After collecting and processing the samples, we compared immunoconversion rates given traditional criteria based on two samples to the rates using criteria based on all three-samples. Further, we explored the data to assess whether there was evidence of simultaneous co-infections (immunoconversions to two or more pathogens). Co-infections have been shown to aggravate and exacerbate the symptoms of AGI-related diarrhea (17, 18).

## Materials and Methods

### Reagents

5.6 micron polystyrene microsphere (bead) sets were obtained from Luminex Corp. (Austin, TX, USA) at a concentration of 12.5 × 10^6^ beads/ ml each. Biotinylated goat anti-human IgG (λ) secondary detection antibody was obtained from KPL (Gaithersburg, MD, USA). Antigens were purchased as described and coupled to the beads in accordance with the optimized multiplex immunoassay (5, 8). Characterized sera was obtained from SeraCare (Milford, MA, USA) to validate the assays.

### Antigen Coupling and Confirmation Using Animal-Derived Antibodies

Beads were activated and coupled, as previously described (5, 8) and serial dilutions of primary capture antibodies specific to each antigen were used to confirm that the beads were coupled properly thus ensuring that the dynamic range of the assay could be defined (5, 8). Briefly, coupled bead stocks were diluted in PBS-1% BSA to a final concentration of 100 beads/μl of each unique bead set. 5 × 10^3^ beads from each bead set was added to individual wells of a pre-wet 96-well filter plate. An equal volume 2-fold serial dilutions of anti-species IgG primary antibody was added to the beads, mixed gently, covered, and allowed to incubate in the dark, at room temperature for 30 min at 500 rpm on a VWR™ microplate shaker (Radnor, PA, USA).

After incubation, supernatant was vacuumed out, wells were washed twice with 100 μl of PBS pH 7.4 containing 0.05% Tween 20 (PBS-T) (Sigma, St. Louis, MO, USA) and vacuumed again to remove excess buffer. Beads were resuspended in PBS-1% BSA buffer and exposed to 0.8 μg of biotinylated anti-species IgG secondary detection antibody. The filter plates were covered and allowed to incubate in the dark at room temperature for 30 min on a plate shaker. After a 30-min incubation in the dark on a plate shaker to protect the beads from bleaching, the wells were washed twice as above. Then the samples were incubated for 30 min with 1.2 μg of streptavidin-R-phycoerythrin, vacuumed, washed X2, and resuspended in 100 μl of PBS-1% BSA. The plates were then analyzed on a Luminex 100 analyzer (Luminex Corporation, Austin, TX, USA).

### Saliva Collection, Processing, and Analysis

Saliva samples were collected from 2,091 study participants at Boquerón Beach, Puerto Rico during the summer of 2009 as part of the National Epidemiologic and Environmental Assessment of Recreational Water Study (NEEAR) conducted by the United States Environmental Protection Agency (19). A key criterion for selecting the beach was ensuring that local residents were the primary visitors; hence, while tourists do recreate at the beach, visitors are largely locals with over 70% indicating six or more visits to the beach annually. Informed consent was obtained from subjects in accordance with Institutional Review Board approval (IRB # 08-1844, University of North Carolina, Chapel Hill, NC, USA). Study participants were instructed to rub the Oracol™ saliva collection device (Malvern Medical Developments, Worcester, U.K) against the gingival crevices of the oral mucosa (between the gums and teeth) to absorb saliva. Infants younger than 1 year old were excluded from the study because of the potential for contamination by maternal antibodies and high rates of non-waterborne infections. Individuals who reported dental or other illnesses at the time of the initial collection were also excluded.

The baseline samples (S1) saliva samples were collected on the beach by trained study staff members while the second and third samples were self-collected by the participants at home and within a day or two post-collection, the samples were shipped overnight on ice to USEPA in Cincinnati for storage and analysis. Upon receipt, the swabs were stored at −80°C until ready for processing which typically occurred in <1 week. The Oracol™ saliva collection devices were thawed to room temperature and centrifuged twice (first at 491 × g, 10°C for 5 min to recover the saliva off the collection sponge and then at 1,363 × g, 10°C for an additional 5 min to pellet debris from the saliva) and transferred to 1.5 ml microcentrifuge tubes. Finally, the samples were centrifuged at 1,500 × g for 3 min and the supernatant transferred to a fresh 1.5 ml microcentrifuge tube and stored at −80°C.

A 1:4 dilution of the saliva samples in phosphate buffered saline containing 1% bovine serum albumin (PBS-1% BSA) was added to prewet and vacuumed 96-well filter plates (Millipore, Billerica, MA, USA). 5 × 10^3^ beads from each bead set and an equal volume of diluted saliva were loaded onto each well resulting in a final dilution of 1:8 in a total volume of 100 μl per well. The loaded filter plates were processed, as previously described (5, 8), reporter fluorescence was measured using a Luminex 100 analyzer and expressed as MFI (Median Fluorescence Intensity) of at least 100 beads per bead set.

### Assay Controls, Cross-Reactivity, and Signal to Noise Ratio (SNR)

Assay controls are described in greater detail in previous publications. Briefly, an uncoupled unique bead set was added to the assay to evaluate non-specific binding and sample to sample variability (5, 8). These control beads were treated identically to antigen conjugated beads and blocked with BSA. However, they were not coupled to any antigen during the coupling step. To control for non-specific binding and/or contamination of the saliva by serum from gum disease or other sources, samples with reactivity to uncoupled control beads at ≥500 MFI were discarded.

Tests for cross-reactivity were performed in monoplex, duplex, and multiplex for each antigen. Assay sensitivity was validated with characterized human plasma samples as previously described (8). A signal to noise ratio (SNR) was calculated by dividing the MFI of the specific antigen signals by the MFI of the uncoupled control beads for each sample (8, 20).

### Defining Immunoconversions and Data Analyses

Only study participants who gave all three samples were considered and two definitions, or immunoconversion criteria (IC) were evaluated. Criteria A is based on the commonly used approach for determining seroconversions for clinical samples collected in a controlled environment: a 4-fold MFI increase from S1 to S2 (S2 ≥ 4 × S1) (14, 21, 22). The second criterion (Criteria B) was developed by extending the traditional definition to ensure that the S2 sample MFI is immunopositive [greater than the cut-off point (9)]; and the S3 sample MFI is at least three times that of the S1 sample (S2 ≥ 4 × S1; S2 ≥ cutoff; S3 ≥ 3 × S1). To reduce potential false positives, we measured antibody responses in the S3 sample. IgG levels are expected to remain relatively high and not drop to zero during the 30–40 day period after initial exposure for all the organisms in this assay. The addition of the S3 sample may guard against considerable variation in volume and composition as well as possible errors in labeling since S2 and S3 were self-collected. All data analyses were performed using Microsoft Excel 2016, JMP 14, and MATLAB Release 2018b.

## Results

### Study Population

**Figure 1:**
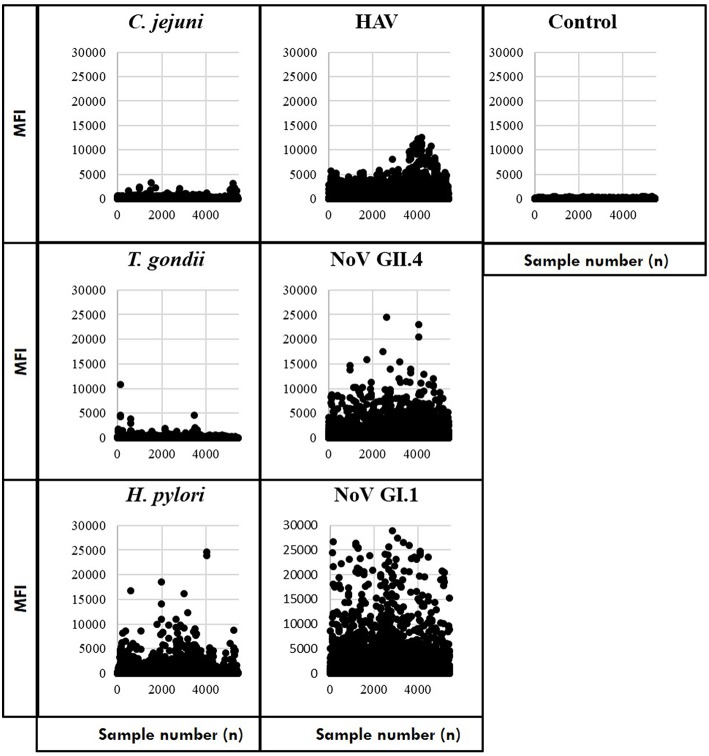
Median fluorescence intensity (MFI) for the target pathogens and assay controls.

Study participants provided 5,533 serially collected samples which were processed to prepare the immunoassay results for statistical analyses (Supplementary Material). Before assessing immunoconversions, we removed samples with control bead MFI ≥500 (as previously noted) and computed a mean MFI of samples with duplicate IDs. The remaining samples included saliva collected from 2,078 individuals at the beach (S1) and then self-collected by 1,694 individuals at S2 and 1,666 individuals at S3. Scatterplots of the MFI results suggest that IgG antibodies to the noroviruses were more prevalent (the most frequently detected of the targeted pathogens) in the study population followed by hepatitis A virus (HAV) and *H. pylori* (Figure 1). In contrast, most of the *T. gondii* and *C. jejuni* MFI results were near the level of the control beads. Of these samples, MFI results were available from 1,399 individuals who provided saliva during all three collection periods. This cohort was used to estimate the number of immunoconversions by measuring IgG antibody levels against the six antigens in the Luminex multiplex immunoassay and computing immunoconversions based on the criteria developed.

### Immunopositivity and Immunoconversions

**Figure 2:**
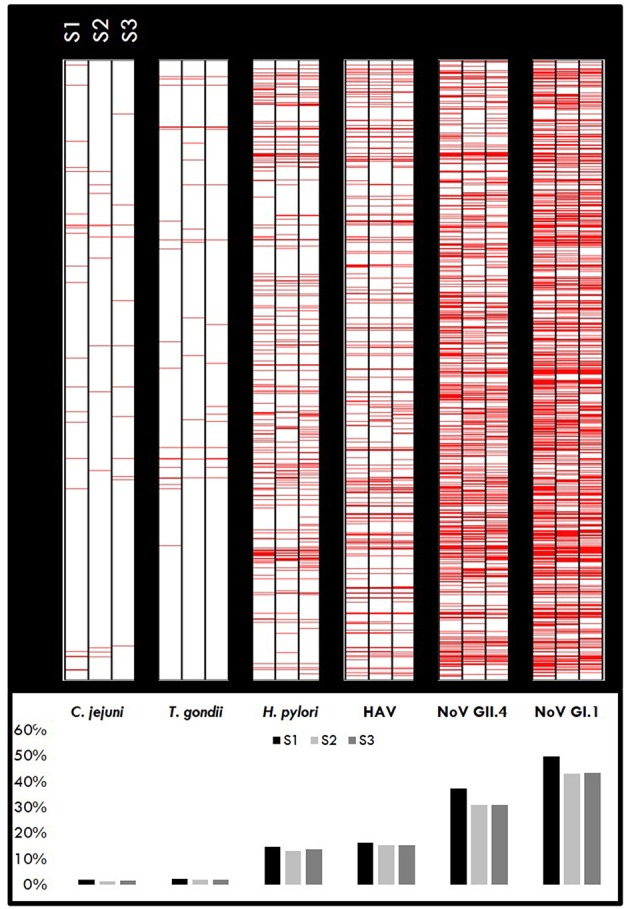
Immunopositivity heatmaps showing MFI response by pathogen for individuals who gave all three samples (*n* = 1,399). Upper panel: Red lines represent positive samples (≥ cut-off) for S1, S2, and S3 samples for each pathogen. The darker areas indicate a higher number of positive samples. Lower panel: Percentage of samples positive for each pathogen.

A cut-off value of 505 [criteria = 10^mean(h)+3SD(h)^, where *h* = log (control) and SD = standard deviation] was previously developed as a part of the Boquerón immunoprevalence study (9); hence, samples with MFI ≥505 for the study pathogens are considered immunopositive. A heatmap for the target pathogens (Figure 2) provides a visualization of the immunopositive samples (in red) at each collection time point (Figure 2, upper panel). Results indicate that over 60% (68, 61, and 62% in S1, S2, and S3, respectively) of the individuals were immunopositive to at least one pathogen during each sample period. As expected, the cohort of beachgoers was primarily exposed to noroviruses (NoV GI.1: 45.34% and NoV GII.4: 30.05%) followed by HAV (15.68%; reported as a mean over the sample periods) and the highest levels were found in the baseline sample (S1), indicating that these participants were exposed prior to providing the initial sample on the beach.

MFI trends reflect antibody response patterns and afford the ability to estimate immunoconversions in the study population. When comparing the two immunoconversion definitions, there were 363 (25.9%) and 91 (6.5%) of the 1,399 individuals who immunoconverted to at least one pathogen, given Criteria A and Criteria B, respectively. For Criteria A, the immunoconversions amongst the specific pathogens ranged from 97 (6.93%) for NoV GII.4 to 166 (11.87%) for *T. gondii*. Conversely, there were much fewer immunoconversions based on Criteria B with most being to noroviruses (NoV GI.1: 39(2.79%); NoV GII.4: 36(2.57%) followed by *T. gondii* (22(1.57%) (Table 1). While most of the immunoconversions were to one pathogen, many individuals immunoconverted to multiple simultaneously (Criteria A: 46.56 %; Criteria B: 24.18 %).

**Table 1:** Immunoconversions to target pathogens: number (*n*) and percentage (%) of individuals that immunoconverted.

**To Single and Multiple Pathogens:** ***n*** **(%)**		
**IC**	**Criteria A**	**Criteria B**
Single (*N* = 1)	194 (53.44)	69 (75.82)
Multiple (*N* ≥ 2)	169 (46.56)	22 (24.18)
**To Specific Pathogens: n (%)**		
**Pathogen**	**Criteria A**	**Criteria B**
*Campylobacter jejuni*	107 (7.65)	1 (0.07)
*Helicobacter pylori*	127 (9.08)	2 (0.14)
*Toxoplasma gondii*	166 (11.87)	22 (1.57)
Hepatitis A virus	99 (7.08)	20 (1.43)
Norovirus GI.1	121 (8.65)	39 (2.79)
Norovirus GII.4	97 (6.93)	36 (2.57)

Results from the less stringent criteria (Criteria A) reflected multiple simultaneous immunoconversions to all the pathogens in the study. Although several of the samples were found to meet the 4-fold increase guideline between S1 and S2, many of these samples never exceeded the immunopositivity cut-off level (MFI ≥505). Therefore, these samples were considered immunonegative. Accordingly, we shifted away from Criteria A and used the more stringent definition (Criteria B) which not only assures that the second sample is immunopositive, but by including S3, also affords the ability to capture antibody patterns that mimic the anamnestic response and could help reduce the number of false positives.

**Figure 3:**
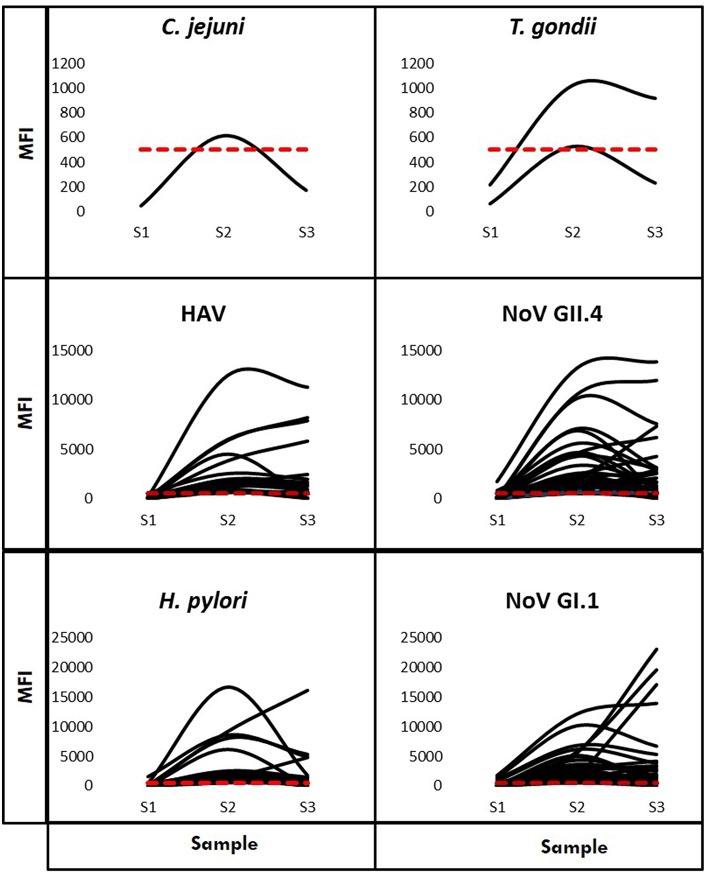
IgG antibody response curves for the six multiplexed pathogens (Criteria B). Characteristic curves showing antibody responses measured in MFI from the baseline (S1) to the final (S3) sample for individuals who immunoconverted. The red dashed line represents the cut-off point (MFI = 505).

**Figure 4:**
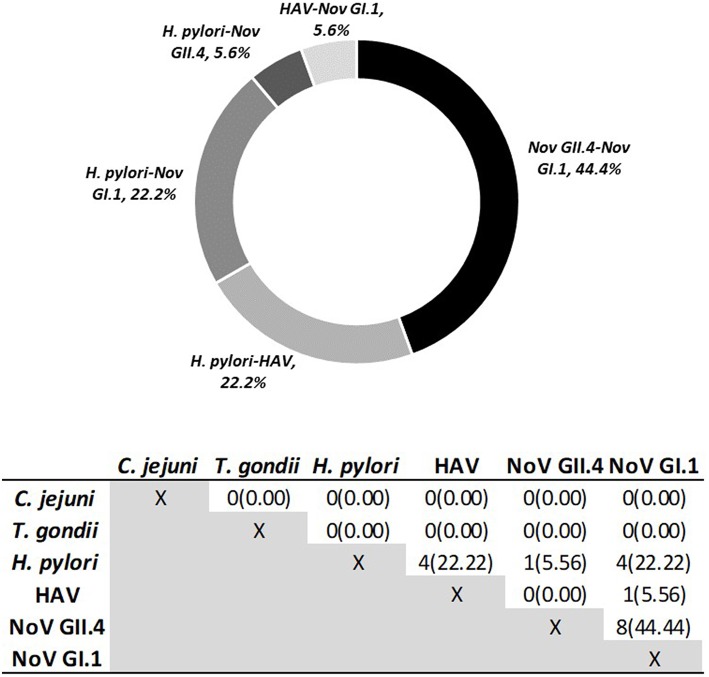
Co-Immunoconversions to specific pathogen pairs calculated using Criteria B. Number of individuals (*n*) who immunoconverted to both pathogens simultaneously.

Using Criteria B to further examine immunoconversions, we note that 69 individuals (75.82%) immunoconverted to one pathogen and 22 individuals immunoconverted to multiple (24.18%), resulting in 120 total immunoconversions. Figure 3 provides IgG antibody response curves for the immunoconversions captured during the study period. In the plots, the dashed red line represents the immunopositivity cut-off value (MFI = 505) and helps to highlight the number of ways individuals immunoconverted to the target pathogens based on Criteria B. All immunoconversions met the core criteria (S2 ≥ 4 × S1; S2 ≥ cutoff; S3 ≥ 3 × S1), yet a small number of them (*n* = 9) presented with immunopositive baseline samples indicating previous exposures, mostly to noroviruses. In addition, rather than declining from S2 to S3, some of the MFIs continued to increase suggesting the possibility of new exposures at S2. Of the immunoconversions to multiple pathogens, the majority (*n* = 18) were to only two pathogens. Figure 4 provides a breakdown of the co-immunoconversions observed by specific pathogen pairs. Nearly half of these co-immunoconversions were to the noroviruses (44.4%) and over a fifth were to *H. pylori* and Nov GI.1 (22.2%) and *H. pylori* and hepatitis A (22.2%).

## Discussion

Detecting exposure and incident infections to environmental pathogens has long been a complex environmental and public health challenge. Classic approaches involve epidemiological studies and surveys capturing demographic and symptomology information, collection of water, soil, and/or air samples to detect organisms in media and complex modeling to estimate potential impacts on human populations. As an augmentation of this practice, this effort extends the use of salivary antibodies as biomarkers of exposure. Rather than estimating the risk of exposure, our approach relies on the body's immunological response to determine whether exposure and subsequent infection to specific pathogens has occurred. Capitalizing on the availability of antibodies in saliva samples, we developed a multiplex immunoassay to measure IgG antibodies against waterborne pathogens. The optimized assay was used to estimate immunoprevalence to targeted pathogens given saliva samples collected from beachgoers at Boquerón Beach, Puerto Rico (9). Additionally, we identified a breakthrough connection between asymptomatic norovirus infections and swimming at the beach (impacted by sewage from publicly-owned treatment works) finding that infected parties did not necessarily present with gastrointestinal symptoms (15). The assay was also applied in a prospective community study of waterborne infections to characterize associations between water-related exposures, *Cryptosporidium* and norovirus infections and gastrointestinal symptoms (6).

In this work, we applied the multiplex immunoassay to investigate antibody responses and detect evidence of immunoconversions to six target pathogens in visitors at Boquerón beach, Puerto Rico. Our effort was a blind examination of the study population focused on IgG antibodies characterized by patterns in median fluorescence intensity and aimed at providing an immunological response screening approach for all pathogens in the multiplex.

After applying the immunoassay and processing the MFI results, we found that 1,399 visitors provided samples during all three collection periods and on average, 892 (64%) of them displayed evidence of detectable antibodies to at least one pathogen (9). This is consistent with previous studies of acute gastrointestinal illness (AGI) performed in multiplex (23–26). These observations demonstrate the potential for multiplex assays to identify multiple infections and may be applied either cross-sectionally or longitudinally to provide insight into the epidemiology of these conditions, as well as understanding the role of potential risk factors.

When only a single pre- and post-exposure sample is available, calculating immunoconversion is typically based on a 4-fold increase in antibody titer between the initial and follow-up sample (14, 21, 22). This may be suitable when the samples are collected in controlled clinical settings; however, collection inconsistencies arise when samples are collected in a field setting where antibody responses in individuals may be more variable due to factors related to self-collection (e.g., sample contamination, spitting). While many individuals demonstrated typical immunoconversions, with a 4-fold increase in antibody levels that was sustained 6 weeks later (S2), we noticed some variations in the data. Examples of this variability included cases in which: (1) we found a 4-fold increase in antibody levels from S1 to S2 but the MFI during both periods was below the cut-off point (MFI = 505); hence, neither sample was immunopositive, or (2) cases where the baseline sample (S1) level was already above the immunopositivity threshold, suggesting previous exposure, and the individual did not experience a 4-fold increase MFI for the targeted pathogen at S2. Using the traditional approach (Criteria A), we found 363 (25.9%) immunoconverted individuals compared to 91 (6.5%), resulting in an almost 4-fold decrease when using the more stringent definition (Criteria B).

In our study, some of the saliva samples were self-collected and varied considerably in quality, volume, and composition. We used the follow up sample (S3) to more fully characterize the immunological response of individuals who showed the initial increase in antibody levels and account for the variability associated with the saliva samples. The addition of the third sample afforded the ability to examine patterns reflective of an anamnestic response more tightly linking the immunoconversion criteria (Criteria B) to expected immunological patterns. The more stringent immunoconversion criteria (Criteria B) may present the best option to analyze the data because, in most cases, IgG responses are expected to remain elevated for months to years, in some cases, following initial exposure and infection (27–32). As a result, the third sample may protect against potential false positives resulting from sample to sample variability in antibody concentrations due to collection technique or volume of sample collected. Further, immunoconversions to pathogens with relatively long incubation periods (for example, HAV−19–49 days) may not be detected using the two-sample approach with S2 being collected about 10 days after the initial sample.

As noted in Wade et al. (15), the number of participants who reported symptoms of AGI was much lower than expected. The low number of symptomatic individuals may be attributed to the fact that many of these individuals had been exposed to those pathogens previously and therefore were immunologically protected and accordingly, did not exhibit symptoms (e.g., diarrhea, vomiting). We observed evidence of protective immunity during our analysis of immunoprevalence where participants were found to have very high antibody levels in the baseline (S1) samples which were indicative of prior exposures (9) most without complaints of the symptoms of gastrointestinal illness (15). This phenomenon of high underlying immunity in a population is not uncommon and as a recent example, was observed during an outbreak of hepatitis E virus in Chad (33).

There are several limitations to our study. Firstly, although antibody binding appears to be specific within the context of these antigens, we cannot eliminate the possibility of cross-reactivity. The issue of cross-reactivity (binding to similar or overlapping ligands) or multi specificity (binding of distinctly different ligands or different conformations of the same antibody (34–37) continue to plague immunological studies. Questions pertaining to non-specific binding and cross-reactivity were addressed by using PVX buffer vs. PBSA (8, 9, 38). Our analysis showed that PVX buffer was superior to PBSA in reducing non-specific binding in plasma and serum samples but had very little effect on saliva samples (9).

Another limitation of the study is that antibody levels in saliva samples are typically lower than levels observed in sera or plasma (36). Some individuals with low specific serum IgG will have even lower saliva IgG concentrations and therefore, these individuals may not be considered when reporting immunoprevalence or immunoconversions. The implication of these findings is that immunoprevalence and immunoconversions may be underestimated by oral fluid IgG detection because the assay may not have sufficient sensitivity when the antibody titer range is low.

Although the immunoglobulin concentration in crevicular saliva is lower than that found in serum, we used the Oracol sampler because it collects crevicular fluid enriched with serum antibodies. In this study, we focused on systemic rather than on mucosal immune responses particularly in the IgG isotypes. It was previously shown that IgA antibody measurement in saliva was unreliable and influenced by several factors including donor's age, secretory flow rate and acute or chronic stresses (39). Furthermore, it has been shown that the proportion of specific to total IgG is similar in saliva and serum (40).

Finally, the ability to separate specific environmental exposures (i.e., at the beach) from other exposures, including those leading to underlying immunity in the population is an ongoing public health challenge. Many pathogens are not solely waterborne, and humans are constantly exposed to potentially harmful agents through what they eat, breathe, touch, etc. Accordingly, it is important to link the findings from exposure studies to occurrence data gathered through water quality studies, soil sampling, air monitoring, etc. to help identify potential sources of exposure for monitoring and management.

Despite these limitations, this multiplex salivary antibody immunoassay has been very useful in measuring immunoprevalence to the six pathogens in the assay as well as identifying asymptomatic norovirus infections in swimmers at Boquerón Beach (9, 15). In September 2017, Hurricane Maria severely devastated the island of Puerto Rico. The hurricane caused significant damage to the island's health, electrical, water, and sewage infrastructure. A comparative study of the post-hurricane immunoprevalence and incident infections would be highly desirable. Such a study would improve our understanding of the impact of natural disasters in the pathogenesis of infections and disease as well as inform risk assessment and management in the aftermath of these events. This assay is being deployed in large epidemiological studies of fresh water rivers, lakes and wells and marine beaches to study the impact of water quality on human health. The results being obtained from these studies will help to improve our understanding of the transmission of environmental pathogens.

The salivary antibody multiplex immunoassay presented here can measure the presence of human salivary antibodies to multiple antigens simultaneously to determine immunoprevalence and immunoconversions in individuals. Furthermore, this assay can identify simultaneous infections and co-immunoconversions in individuals exposed to environmental pathogens. Recreational water sources are typically contaminated with multiple pathogens and hazardous chemicals, each of which can assault the health of exposed individuals simultaneously.

Very little information is available about how these simultaneous infections affect human health and whether they result in synergistic or antagonistic interactions. Griffiths et al. studied the impact of co-infections in humans and concluded that they have a deleterious effect on health exacerbating infection outcomes in humans (41). Moreover, Bhavani et al. observed synergistic effects between rotavirus and co-infecting pathogens on diarrheal disease, noting that the pathogenic potential of each organism appeared to be enhanced during co-infection (42).

We have applied this immunoassay in epidemiological studies at several locations across the United States and were able to estimate immunoprevalence to environmental pathogens, identify asymptomatic norovirus infections, and demonstrate associations between water-related exposures, AGI symptoms and *Cryptosporidium* and norovirus infections. When used in conjunction with water quality studies, this assay can potentially enhance our knowledge and understanding of environmental microbial pathogenesis and assist risk assessment modelers in estimating exposure potential thus facilitating the development of disease surveillance and screening tools thereby leading to better health outcomes and a cleaner, more sustainable environment for the general population.

## Data Availability

All datasets generated for this study are included in the manuscript/Supplementary Files.

## Ethics Statement

Approval was obtained from the University of North Carolina, Chapel Hill, NC, USA (IRB # 08-1844), for the collection of saliva samples from beachgoers at Boquerón Beach, Puerto Rico, as part of the USEPA NEEAR Water Study. Study subjects provided informed consent and were instructed on the use of the Oracol™ saliva collection device. Infants younger than 1 year were not included. Informed consent was obtained from parents of minors.

## Author Contributions

SA designed the study with KS and TE. TW, KO, AD, and ES provided the NEEAR saliva samples. SA, KS, SG, CC, and MR conducted the laboratory experiments and processed the raw assay data. TE performed the data analysis. KS, TE, and SA wrote the original manuscript and KS, TE, SA, CC, SG, MR, KO, ES, TW, AG, and AD participated in the review and discussion of the study data and the criteria development.

### Conflict of Interest Statement

The authors declare that the research was conducted in the absence of any commercial or financial relationships that could be construed as a potential conflict of interest. The reviewer JS declared a past co-authorship with several of the authors TW and AD to the handling editor.
